# Collaboration for new therapies: maximizing health and innovation

**DOI:** 10.3389/fpubh.2024.1383107

**Published:** 2024-09-19

**Authors:** Jorge Mestre-Ferrandiz, Pierre Meulien, Dennis A. Ostwald, Virginia Acha

**Affiliations:** ^1^Department of Economics, Universidad Carlos III, Madrid, Spain; ^2^Life Science Consultant, Faro, Portugal; ^3^Founder and CEO of WifOR Institute, Darmstadt, Germany; ^4^Global Regulatory Policy & Intelligence, MSD, London, United Kingdom

**Keywords:** innovation ecosystem, life sciences sector, funding, incentives, research and development

## Abstract

**Introduction:**

Innovative medicines and vaccines can provide direct health benefits to patients and populations by preventing, treating and curing diseases, and also drive wider socioeconomic and productivity gains. However, researching and developing them is complex and risky. Funding for life sciences R&D has different sources: public, charitable/NGO, and private sector. We believe there is consensus that all are required, although there is less understanding about their respective roles, synergies, and funding priorities. The purpose of this paper is to provide an overview of the current life sciences innovation ecosystem in Europe, highlighting challenges for funding and innovation of new therapies and our proposed options to address these.

**Methods:**

The basis of this paper stems from the reflections made by the co-authors during a webinar with title “Collaboration for new therapies: maximising funding and innovation,” in March 2023, with further targeted literature reviews.

**Results:**

We identify eight challenges in the European life sciences ecosystem, some closely related, and nine options that we think might be helpful to address them. Each option on its own can have different levels of ‘impact’, but collectively will provide synergies among them, and thus maximize their impact.

**Discussion:**

It is critical to ascertain how the strengths of each actor can be leveraged to bring new medicines/treatments to market, quicker and more efficiently. We need a trusted environment, with strategic collaborations between the public and private sectors, and policy initiatives and incentives should be targeted to strengthen the infrastructure with the aim of fostering such optimal alliances.

## 1 Introduction

Innovative medicines and vaccines can provide direct health benefits to patients and populations by preventing, treating and curing diseases (e.g., reducing mortality, or increasing quality of life), as well as driving wider socioeconomic and productivity gains. The reason for this is improved health triggers economic growth through various means, such as improving labor productivity, savings or investments in education and other forms of human capacity/capital [Schiener et al. (1) and Weber (2)]. For instance, Haaf et al. (3) estimate that in 2019, the Health Economy (which is comprised of the Healthcare Economy, Industrial Health Economy and Services & Support) in EU-27, generated a gross value added (GVA) of €1,375 billion and employed 29.3 million people, representing 11 and 14% of total EU-27 GVA and employment, respectively. To put these percentages into context, the contribution of the EU’s Health Economy is equal to the GVA of Spain and Estonia combined and supports jobs equivalent to the entire labor market of France. Within the Health Economy, innovative medicines and vaccines specifically are only one part of this industry and economic activity, however, a pivotal one to drive improved outcomes; these represent less than a quarter of all healthcare expenditures, as recently estimated by IQVIA (4), for the EU (range between 8 and 24% in 2018 for EU countries), and are often used to replace and reduce further healthcare costs through their therapeutic impact – an overview of examples can be found in Weber (2) and EFPIA (5).

However, researching and developing medicines and vaccines is a complex and risky endeavor. Indeed, most of these investments will not necessarily result in a therapy: only around 1 out of 10 proof of concept trials will result in an approved treatment, and from the earlier pre-clinical target selection stages, the probability is even lower, where only about 3 out of 100 will be successful [see, for instance, SiRM et al. (6), Rennane et al. (7), and Schlander et al. (8)]. For most sectors this would be considered too high a risk for investment. Nonetheless, this is how the R&D process works in the life sciences sector. Moreover, the complexity of therapeutic targets for treatments today can be very different to the medicines available on the market two or three decades ago – where we observed new molecule launches for primary diseases, with relatively high volumes and ‘low’ prices per patients (e.g., statins, H2 antagonists or proton pump inhibitors).

Funding for life sciences R&D has different sources, namely public, charitable/NGO, and private sector funding. We believe there is consensus that both the public/charitable and the private sector are required, although there is less understanding about their respective roles, synergies, and funding priorities. Global pharmaceutical R&D investment has been estimated to be *circa* $300bn in 2020 (6). Big biopharma is its largest funder, with $195bn annually and representing almost two-thirds of the total R&D investment. The top 15 biopharma companies contribute more than half of this. Public-sector (*circa* $65bn) and not-for-profit organizations (*circa* $10bn) account for more than a quarter, and the remaining 10% ($30bn) is attributable to venture capitalists (6). Venture capital investment has however been growing strongly in recent years (at a compound annual growth rate CAGR of 14.2% between 2011 and 2019), albeit starting from a low base. Biopharma’s R&D investment CAGR was 4.1% over the same period, while public sector and not for profit grew at lower rates, at 1.1 and 0.8%, respectively, (6).

Generally, for R&D investments, we see important differences across the shares of the different funding sources at national level. Most countries that invest more than the OECD average of GDP on R&D have a private sector to public sector ratio of over 2.5 to 1; for example, South Korea and the US have a ratio of 3.4 to 1 and 2.5 to 1, respectively, (9). For six European countries, it has been estimated that about two-thirds of investment in pharmaceutical innovation therein stems from the private sector (10). Some countries, however, can still rely proportionately more on public sector investments than private.

Needless to say, we believe in the complementarities and synergies between all sectors, with evidence highlighting the complementarity in medical R&D between public/charitable and private sectors, giving rise to spillovers. For the UK in particular, Sussex et al. (11) estimate that a 1% increase in public biomedical and health research expenditure is associated with a 0.81% increase in private pharmaceutical R&D expenditure. Based on the UK’s R&D expenditure, this spillover effect quantifies into an additional £0.83–£1.07 of private sector R&D spend in the UK for every additional £1 of public research expenditure; 44% of that additional private sector expenditure occurs within 1 year, with the remainder accumulating over decades.

At European level, and similarly to other elements discussed later (VC or other specific funding for startups), there is fragmentation of funding sources for health-related research. From the public side, we have both national funding agencies in each MS, and the EU commission funding programs. On the charity side we have multiple disease specific funders, as well as philanthropic funding – national, EU and global wide – adding complexity, duplication and inefficiency. Finally, private sector funding is by definition company specific apart from joint initiatives like IHI (see Box 1). This very complex situation is full of inefficiencies which if harnessed could make a huge difference.

BOX 1 **The Innovative Medicines Initiative (IMI) – now called Innovative Health Initiative (IHI).**

The IMI is the largest public private partnership in the world, enabling to do things at scale, both in terms of generating great research, and bringing the relevant actors together. These connections have brought real value to the European life sciences innovation ecosystem and remains one of the great models worldwide for connecting those who need to be connected to accelerate innovations. The IMI has not only connected researchers, clinicians, and industrial partners, but as importantly also regulators, patients, and health economists. This true multi-disciplinarity gives the richness and has that end to end integration that is needed for transformative results to be generated.

IMI is indeed an example of how things can change, but more needs to be done. Some limitations include that IMI has not been applied to all biomedical fields nor has it had the goal of including everyone, but for certain things, it really has been a sweet spot for the PPP model. It remains pre-competitive, so most work will benefit the system, rather than an individual entity, region, or university. Two prominent examples include the work on market failures (for AMR), and issues with huge public health importance, like drug safety. Other projects have worked on more scientific black boxes (neurogenerative diseases – Alzheimer’s Disease, dementia, Parkinson’s) that require broad stakeholder collaboration before meaningful results can be turned into innovations impacting society. As a result, new clinical research platforms are being/have been built, to reflect the nature of the connectiveness and infrastructure generated by IMI. The objective is ultimately to transform how we perform translational and clinical research for some more complex issues and therapeutic areas. This has really changed the culture of how we do research and has begun to build the trust that is so important to continue the collaboration and partnership that is so valuable. One issue to still resolve via this partnership is the West vs. East European divide, the latter only representing 2–3% of the IMI/IHI ecosystem.

Medicines and vaccines are an integral part of a healthcare system, interconnected with its other components. However, once novel medicines have been developed, patients face access complexities. The recent technical reports commissioned by the WHO Oslo Medicines Initiative emphasize this complexity. It provides an evidence-informed basis for understanding the challenges and potential policy solutions for achieving better access to and improved affordability of novel, high-priced medicines. See, for example, Ardal et al. (12) for a high-level overview of the issues and the complex dynamics as background to the technical reports. Issues discussed therein include affordability challenges either because of high unit prices or due to the low number of potentially eligible patients, uncertainty around therapies’ long-term effectiveness, high level of unmet needs, and administratively burdensome outcomes-based managed entry agreements. There is also heterogeneity across EU health care systems, in terms of funding levels and priorities, as well as evidence requirements to determine the value of the new treatments.

With this context in mind, the purpose of this paper is to provide an overview of the current life sciences innovation ecosystem in Europe, highlighting the challenges for funding and innovation of new therapies and our proposed options to address these. A key aspect to highlight here, and as supported by a 2024 EU Parliament Report (13), is the natural tension that exists between the European vision to harmonize and synergise across member states while the member states guard preciously their national competency in healthcare delivery – this dichotomy needs to be properly managed if we want to improve Europe’s competitiveness. Before defining solutions, however, the next section defines the components of an innovation ecosystem generally and applies them to the life sciences sector.

## 2 What is an innovation (eco)system?

Granstrand and Holgersson (14) provide a review of definitions of innovation ecosystems and related concepts, to ultimately propose their own synthesized definition. They identified, based on 21 varying definitions, seven themes of definition components: actors, artifacts, collaboration/complements, competition/substitute, activities, institutions, and co-evolution/co-specialization. Nevertheless, there are three recurring entities: actors, artifacts, and institutions, and definitions often place emphasis on collaboration/complements and actors, while less commonly on competition/substitutes and artifacts. With this context, Granstrand and Holgersson (14) define an innovation ecosystem as “the evolving set of actors, activities, and artifacts, and the institutions and relations, including complementary and substitute relations, that are important for the innovative performance of an actor or a population of actors” (pp. 3).

Innovation ecosystems determine, on the one hand, the innovations occurring within their boundaries, the processes that generate innovation and the nature of innovation that results from these systems. On the other hand, innovation systems reflect the range of actors that are present. Thus, how actors are interacting and what mediates that is critical, and particularly, the interface between the agents in the public/charitable and private sector. As Edler and Fagerberg (15) stated, “these systems are more than frameworks for interaction, however, they are also repositories of various resources that firms depend on in their innovation activities and home to various institutions influencing these.” Those resources, often complementary, include knowledge, skills, finance and demand. And these factors, to a large extent, can be regarded as being shaped by the legislative and investment choices of national government, within a broader history of national culture and science.

For the life sciences sector in particular, we argue all seven components identified by Granstrand and Holgersson (14) are pertinent, and indeed some have substantially changed over the last decades. There is now a wider range of actors, and the interactions among them can be more complex. For example, we are increasingly observing partners that would usually be competitive to be complementary, and vice versa - these dynamics, and how they work together, are the ‘secret sauce’ as to why some innovation ecosystems work better than others. Thus, we need to understand these dynamics and synergies in a deeper way if we are to improve the development, funding and access to novel therapies in Europe. Before that, it is important to summarize at high level the five steps required to ultimately ensure the availability and accessibility of effective and innovative treatments for patients:

1. Basic research: which generates scientific knowledge or defines a need.
2. Preclinical and translational research: applies the prior and new knowledge for the development of specific targets that can potentially become new cures and treatments.
3. Clinical research: trials in human beings/patients to test the safety and efficacy of potential treatments.
4. Regulatory approval: provides the marketing authorisation (with sometimes the requirement to generate further evidence).
5. Market access: in many jurisdictions, third party ‘payers’, broadly defined, will determine which medicines are funded, for which patient, and at what cost.

**Figure 1:**
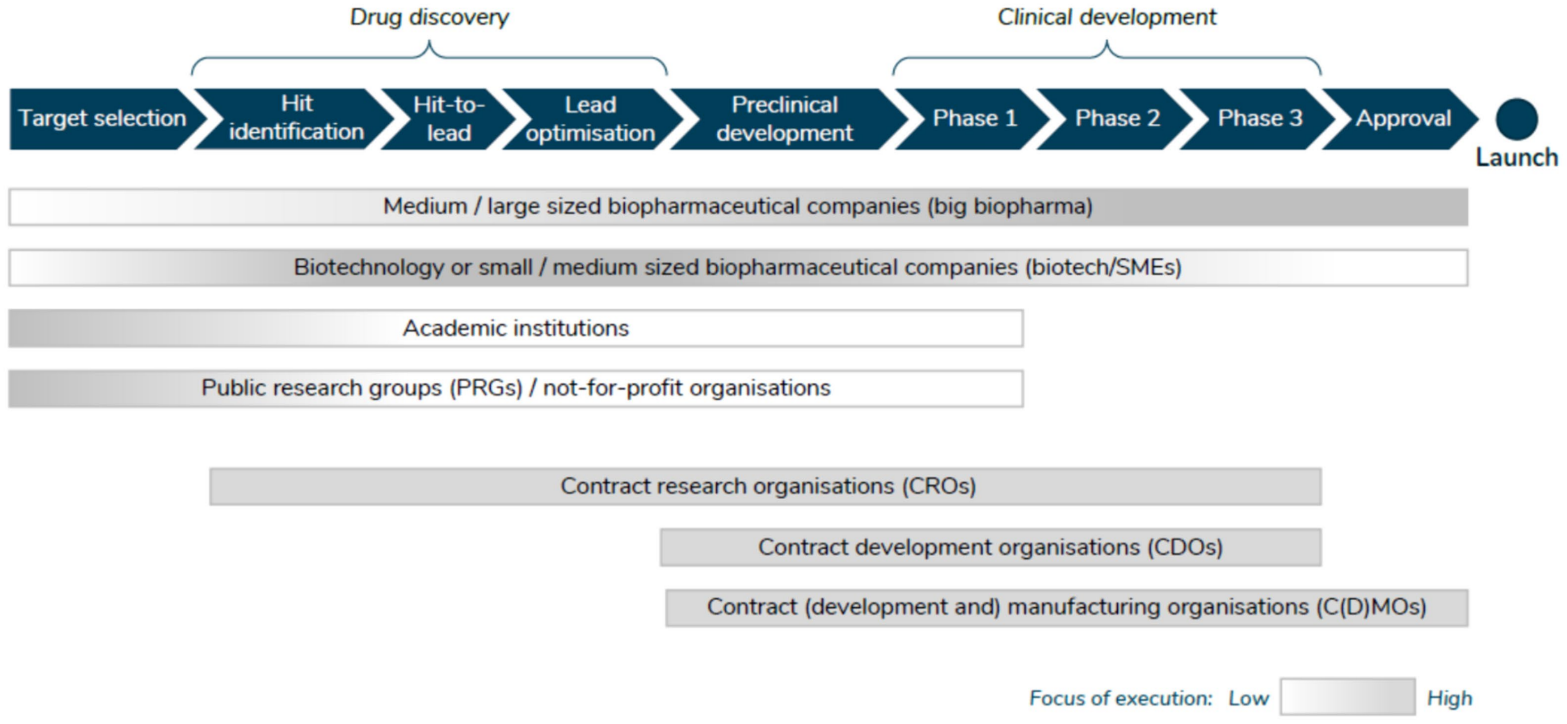
Actors involved in the R&D process. Source: SIRM et al. (6).

SIRM et al. (6) provide an overview of the actors involved in the first four stages above. First, Public Research Groups (PRGs)/not for profit, whose activities are directed primarily toward target selection and drug discovery, with some activity in the early clinical development stages. Second, academic institutions tend to focus on the early stages of drug discovery (target selection and hit identification). Third, big biopharmaceutical companies are involved throughout all clinical development phases, and increasingly are more involved in drug discovery activities (via partnerships and collaborations). Biotechnology companies/SMEs, on the other hand, tend to be more active in the interface between discovery and development, collaborating with different partners along the way. Increasingly, however, these companies are seeking to commercialize their assets themselves and hence are undertaking the latest stages of clinical development Figure 1.

Another important aspect, which is related to various components, is that diversity in funding reflects different ecosystems, including where science is based. The core foundation for the current and future novel therapies will be the life sciences researchers themselves. It is, therefore, important to ascertain why certain universities, or jurisdictions, are stronger than others in this area. Of course, governments (or ‘institutions’ as per one of the components mentioned above) can intentionally influence this process. For example, South Korea has prioritized a dynamic, inclusive and creative economy since 2013, as a part of its effort to secure the growth potential to unlocking the maximum productivity capacity of Korea’s science, technology, innovation and cultural ecosystems (16). Thus, it is not surprising to see the volume of activity occurring there, and it seems there is the ecosystem ‘chemistry’ to create better partnerships and collaborations. More generally around the Asia Pacific region, SIRM et al. (6) find that VC investment is proliferating rapidly, mainly driven by the Asia-Pacific region. Deloitte (17), on the other hand, illustrates how emerging technologies, such as 3D printing and artificial intelligence, can create opportunities for the industry in the region, advocating for new partnerships. Sometimes, the evolution of innovation ecosystems is historic, reflecting change over time. For instance, the case of ‘Oxbridge’ in the UK possibly illustrates this example, given they can be deemed as an ‘old’ ecosystem, but one which has evolved over time.

In Europe, evidence is mounting emphasizing the fragmented nature of Europe’s investment sector as a hurdle to innovation and growth, and the resistance from larger member states to establishing a more integrated system. The evidence also highlights the challenges faced in funding innovation [with a strong emphasis on small and medium-sized enterprises (SMEs)] and the benefits of developing a Europe-wide harmonized and integrated approach to investment and capital markets [European Commission (18), European Investment Bank (EIB) (19)].

Globally, R&D investments in the life sciences sector are increasing, but where those investments are being done is changing. This is argued by Moll (20), who notes they are moving out of the EU: 31% of the total R&D investments made in the US, Europe, China and Japan occurs in Europe, a steady decline from 41% in 2001. China, meanwhile, has grown its share from 1 to 8%. In 2002, the US spent $2 billion more than Europe on R&D; today that difference has grown to $25 billion.

If we look at where clinical trials are being performed (step 3 above), there is a similar trend, with a significant amount of shift in regional utilization with Europe, North America, and China as the most utilized regions that have experienced the largest changes (21). Countries in Western Europe have lost importance between 2019 and 2023, with a 21% decrease in its global share, from 32 to 25%; the loss in share is even more dramatic for Eastern Europe countries, decreasing 33% over the same period (from 17 to 11% market share). On the contrary and following what we observe with general R&D investments, North America’s share has increased by 17% (from 19 to 23%), remaining in second position. China’s share increases by 57% (from 10 to 15%), becoming the third largest region for trial activity, from being fifth in 2019 and overpassing Central and Eastern Europe, and Asia-Pacific. The IQVIA report (20) also classifies countries by patient availability and operational readiness, identifying nine countries as ‘top tier’, five being European (France, Italy, Germany, Spain and the UK), and the rest being China, Japan, South Korea, and the US. Importantly for the future, 11 countries are identified as ‘opportunity tier’, which can become more attractive as a location for clinical trials – all of which are non-European.

Within this context, there is evidence to support the argument that Europe should have a more streamlined system, similar to the United States, for improving competitiveness to attract pharmaceutical investments and clinical trials [EMA (22), EMA (23), EMA (24), European Parliament (25)] and that the clinical trials regulation in Europe has not made the region as competitive as desired [see for instance, European Parliament (25)]. For instance, the EMA’s Regulatory Science Strategy to 2025 includes the goal of harmonizing regulatory processes, reducing administrative burdens, and promoting innovation to attract investments in the European pharmaceutical sector (22). Along similar lines, a European Commission document discussing the compassionate use of medicines – which is a member state competency - highlights the importance of streamlining regulatory processes and reducing administrative burdens to facilitate timely access to innovative therapies in Europe (24).

In terms of market sizes and market shares of medicines (step 5 above), the picture is also changing. During the period 2018–2023 the Brazilian, Chinese and Indian markets grew by 12.3, 5.4 and 9.9%, respectively, compared to an average market growth of 7.4% for the top 5 European Union markets and 8.4% for the US market (26). The US still dominates the global pharmaceutical market, accounting for 53.3% of world pharmaceutical sales compared with 22.7% for Europe. But the difference is even higher when looking at shares of the new medicines launched during the period 2018–2023: the US represents 67% of total sales, compared with 15.8% for the European top-5 markets (26). Furthermore, the origin of the medicines is changing; during the period 2019–2023, nearly 20% of all new chemical and biological entities were from China (according to nationality of other company), while prior to 2019, their importance was negligible (26).

Finally, we also believe the evolution of the life sciences ecosystem is not only linked to resources, and talent, but very importantly, how free are those resources to move between one sector to another. However, it would be (very) difficult to have a flow of resources (including talent) if already there is conflict in the ecosystem. Moreover, we feel there is a younger generation of scientists with a lack of trust and engagement with the private sector, specifically in Europe but less so everywhere else in the world, that will drive the type and nature of collaborations in the future. Evaluating in detail strategies to attract talent are beyond the scope of this paper, but experiences from other sectors can be illuminating to identify potential best practices. For instance, Amazon highlights in their website how they hire and develop the best talent, and studies have shown how attracting talent implies understanding what they want (27) (wider than just financial compensation) and the importance of offering flexible and hybrid work environments and promoting the meaning and purpose behind the work, more than the work itself (28). Berube et al. (29) identified five core themes driving (or hindering) the talent and technology decisions underpinning the success of large-scale digital transformations across a range of industry sectors: plan, hire, train, deploy, and grow which all have to be considered to attract talent.

## 3 Current challenges for funding and innovation of new therapies in Europe

We have identified the following eight challenges in the European life sciences ecosystem for funding and innovation of new therapies, with some of them being closely related:

1. Health is seen as a cost rather than as an investment: it seems the positive momentum about ‘health equals wealth’, and ‘health is an investment’ generated during the COVID-19 pandemic has been lost.
2. Perceptions and expectations, and lack of trust: there is no general consensus about problems and solutions, and a lack of trust among actors, leading to limited collaboration.
3. Need to keep the faith: time is critical for the life sciences sector, given the high uncertainty and unpredictability of medicines’ development process. For the public sector, including universities, the challenge is sustaining enough funding and resources to keep the process moving forward. The private sector requires the right incentives and the right encouragement to continue investments at the level and pace we need.
4. Europe is good at certain parts of the innovation process but fails in others. For example, Europe is good for the creative research and invention, but fails in bringing those inventions to use/market in an efficient manner (i.e., scaling up inventions to become innovations). Complexity of funding schemes is not supporting agile processes particularly for SMEs/biotech, as well as a risk averse private pharmaceutical sector.
5. Poor value proposition: no agreed demonstration of value to the end user and society as a whole of the technologies and services developed.
6. Lack of connection of upstream (early/basic science) with downstream needs (medicines/therapies available and used).
7. Too many silos: there are many actors involved in the development process, including university research, hospitals, and the private sector, where the language might be the same, but the manner of expression and meaning can lead to misunderstandings.
8. Talent shortage: (i) the life sciences sector is global and competitive, and companies decide in which regions to spend their R&D investments according to many factors. Thus, regions and countries can implement policies to attract those investments, especially between the US, Asia and Europe; (ii) versus other sectors, e.g., fintech. The lack of trust among stakeholders (challenge 2 above) can lead to negative perceptions about working in the health industry, which we feel is not the right attitude.

The next section offers our options to address these challenges.

## 4 Options to address these challenges

We have identified nine options that we think might be helpful to address these challenges. Each option on its own can have different levels of ‘impact’, but collectively will be able to provide synergies among them, and thus maximize their impact.

### 4.1 Need to consider (or keep the momentum) of health as an investment, and invest in health smartly: establishing health metrics and measure/quantify them

We feel there is a need for a paradigm shift when measuring the impact of investing in health to realize the societal gains possible from improved population health and the importance of the positive feedback loop within the innovation ecosystem to achieve better health, and growth [G20 (30)]. This is particularly related to economic policy-making in the current context, with the post-pandemic recovery, inflationary pressures, and widening inequality.

At patient level, the use of HTA is the traditional tool to measure ‘impact’ (or effect) of new medicines. There are numerous ways to carry out HTA assessments, and several organizations have also published their own recommendations, however, there is no consensus on the most appropriate methods, as all have certain strengths and limitations.

At a macro level, Schiener et al. (1) provide the ‘Social Impact Approach’ to quantify the effect of novel medicines, in terms of reducing the socioeconomic cost/burden of illness and create positive effect on society, with the aim of enriching the existing health economic evaluations by adding a macroeconomic perspective to the value assessment of medicines. Similarly the inclusion of productivity impact in health economic evaluations, the Social Impact approach measures gain through avoided productivity losses as a result of a medical innovation. This gain is expressed in monetary terms to enable comparability. Measuring disease burden can then help to direct investments toward areas which generate the most significant outcomes and can support stakeholder’s understanding of the impact of a medical innovation. The work around additional elements of value such as value of hope or insurance value, that could be considered when assessing medicines, beyond the more traditional ones such as added therapeutic value and cost effectiveness [see for instance Lakdawalla et al. (31)], are also relevant here. With this in mind, we could agree on a set of standard metrics to holistically assess the return on health investments through the outcomes delivered to societies and economies.

### 4.2 Make health care systems more efficient, including focusing more on prevention, screening and diagnostics

The issue of ‘inefficiencies’ in health care spending has also been raised, for example, by OECD, where they found that around one-fifth of health expenditure makes minimal or no contribution to good health outcomes (32). Three types of waste are identified: clinical care, operational, and governance-related; recommendations to improve this situation include stopping activities with no value, seeking price competition across alternatives, encouraging use of generics, developing advanced roles for nurses or ensuring patients who do not require hospital are treated in less resource-consuming settings (32).

Three key, interrelated, areas that are imperative, we think, to improve the overall efficiency of the system but which have been somehow neglected are prevention, screening and diagnostics. The OECD mentions ‘prevention’ in its 2017 report (32) in two instances: effective and accessible primary care generally prevents Hospitalisations for Ambulatory Care Sensitive Conditions (ACSCs) and correcting preventable medical mistakes or infections. Screening is mentioned once for cancer in particular – but as a common instance of overdiagnosis or overtreatment. Diagnostic testing is mentioned twice; once also as a wasteful clinical care element, when repeated diagnostic tests or services are undertaken because information is not shared across providers, and secondly as having a positive impact in terms of helping clinicians target their antibiotic use.

It is beyond the scope of this paper to look at reasons for this lack of uptake of preventive measures, screening, diagnostic tests and vaccination. As an illustration, Levine et al. (33) provide some interesting insights as to why there is underutilisation of preventive services in the US, which is “largely the result of an implementation gap rather than an information gap… financial incentives do not align with a focus on preventing chronic and infectious diseases. Currently, most providers, including hospitals and physicians, are paid to treat rather than to prevent disease.” In the UK, in 2018 the Department of Health and Social Care published the vision “Prevention is better than cure,” arguing among other things, that “business as usual,” in terms of investing in the same service models of the past, needed to change, including the focus and spend toward more prevention, and not just cure (34).

Regarding the use of screening and diagnostics, there seems to be mixed evidence about the appropriateness of their current use. However, screening is a key element underpinning the four pillars [(i) prevention, (ii) early detection, (iii) diagnosis and treatment, and (iv) quality of life of cancer patients and survivors] in the recent Europe’s Beating Cancer Plan (35). The Plan clearly states that early detection through screening offers the best chance of beating cancer and saving lives – but that many programs have not been fully implemented, and unacceptable inequalities persist within and between Member States.

For diagnostics, as an example, The Kings Fund (36) reiterates the importance of good diagnostic use (arguing, for instance, that more than 85% of patients seeking NHS care in the UK require diagnostics, that prompt diagnosis can save lives, time and money, and avoids worsening patient outcomes if the wrong treatment is pursued), but finds that diagnostic activity still remains below target in the UK and advocate for diagnostics to become a national priority. In this work, it is also explicitly highlighted that diagnostics have an important role to play in preventive health by improving early detection of illness. Of course, the impact of a good and prompt diagnosis will be reduced when the referral does not work, and patients “get lost in the system” – see for example, Horfmarcher et al. (37) for some evidence regarding treatment rates in lung cancer. On the other hand, Muskens et al. (38) findings, based on a systematic literature review examining the prevalence of diagnostic testing overuse, “suggest that substantial overuse of diagnostic testing is present with wide variation in overuse.” Preoperative testing and imaging for non-specific low back pain are the most frequently identified low-value diagnostic tests.

In addition to the use of diagnostics in screening, evolution in the sensitivity and selectivity of diagnostics is essential for the ongoing progress and use of novel treatments. Our understanding of human and disease biology is the basis for the long-awaited and evolving personalized medicine paradigm that ensures that interventions provided are the ‘right medicine, at the right time for the right patient’. Achieving this goal will contribute substantially to healthcare efficiency.

### 4.3 Need to showcase the impact of the sector, the benefits of working in the life sciences sector, and improve overall the value proposition

There are variations in terms of metrics used by the different stakeholders to drive their decisions and to measure impact, and in particular, between the public and the private sector. The latter tends to rely on return on investment as a metric, while the former might be less accustomed to use that metric to drive decisions. Nevertheless, quantifying the impact is of limited value if not communicated correctly to the appropriate stakeholders. Indeed, the number of stakeholders involved in pharmaceutical and health care policy have been increasing over time and their ‘language’ and expertise differs. For this reason, we believe the public needs to better understand that investing (wisely) in health creates value to individuals and society. The examples discussed in the Introduction about the economic impact of the sector, both in terms of GDP and employment, could be used to showcase the sector’s importance.

We suggest a collaborative model across the various stakeholders can lead to better articulation of value of health research and technology but this might imply getting out of their comfort zone to change behaviors.

We also believe that showcasing the positive impact of the sector can help attract the young people needed in Europe. Perhaps we can use the shift to and focus on quality of life after the pandemic to communicate the purpose of working in the sector, having the possibility to change things and to collaborate with people and innovative technologies.

### 4.4 Need to develop strategic public private partnerships

We advocate for a truly strategic public private collaborative model, not just about specific projects, but rather, about new thinking and ways of working together, from early research to manufacturing, to drive better health for all. Governments have a critical role to play to create a more efficient ecosystem that enables more interaction between public and private actors, and supports innovations being scaled up. Generating a positive return on health investments might uncover some fiscal space to increase investment in health.

We would like to mention two examples of instruments from the public sector that have recently sought collaborative private sector funding. Firstly, the European Innovation Council (EIC), launched as a pilot in 2018, has been established under the EU Horizon Europe programme. According to the information on its website^1^, it has a budget of €10.1 billion to support game changing innovations throughout the lifecycle from early-stage research, to proof of concept, technology transfer, and the financing and scale up of start-ups and SMEs. Interestingly, in the last few years the EIC started to mix the funding, both from public and private/equity funds. Secondly, the EIT Health^2^ and the European Investment Fund (EIF) are partnering to operate the Venture Centre of Excellence (VCOE)^3^, a public-private co-investment programme to empower finance for European health SMEs. It is aimed for SMEs that are seeking more than €6 million in their next fundraising round, given the difficulty in finding investors for over €5 million.

Two examples of strategic partnerships at European level are The Innovative Medicines Initiative (IMI)^4^ and the WHO Oslo Medicines Initiative, now transformed to the WHO Novel Medicines Platform (NMP)^5^. The latter is an initiative led by WHO on access to medicines, by bringing together all actors, including the life sciences industry. Box 1 has specific information on the former, IMI, describing the impact such public private partnership has had since its inception.

### 4.5 Building trust across public and private actors through increased transparency about how these different actors collaborate and function

Perhaps one of the lessons from the pandemic is that the public expects quick responses in any key global health crisis. But unfortunately, it could be argued the agents involved in the research and development process did not explain how innovation happens in this sector, that it is not a single event, but rather a practice over decades. Indeed, some of those innovations against COVID-19 were not *de novo*, and we had some options on the shelf to be deployed. As private innovators, there is a need to explain better how the private life sciences sector works, and how they partner and engage with public sector researchers. It seems for too long it was assumed no one needed to know how material transfers agreements or licensing deals work, for example. But the private sector supports the public sector, at least via the following two mechanisms:

1. Financially, the private sector can provide investments to create infrastructure and cover overheads from public sector organizations, including universities and hospitals and any drug discovery units they might have within them. For earlier stages of research, strategic partnerships/collaborations with the private sector allow universities to cover the full economic cost; this is particularly important as many publicly funded research grants usually cover around 80% of these costs. For the later clinical studies, (public) hospitals running them in collaboration with the industry can cover hospitals’ overheads, as well as providing treatments at no cost to patients. Indeed, SIRM et al. (6) find an overwhelming proportion of private funding of the larger later stage clinical trials (relative to the earlier invitro or smaller proof of concept studies).
2. Non-financial, via human capital and knowledge transfer from the private sector to ‘academic’ units, and vice versa, can help agents be better and more efficient at medicines discovery and development. We have already argued that movement of talent across sectors is essential for an innovation ecosystem.

### 4.6 Private sector responds to increased market certainty and incentives: use price as a signal to direct its research

The private sector also faces difficult challenges, given their finite resources to address many unmet medical needs, the uncertainties surrounding the R&D process and the potential returns if successful. We believe the private pharmaceutical sector responds to market certainty and incentives, as it tells innovators where to put their energy and efforts. For this reason, we need to translate public sector needs into signals that work for innovators, on topics deserving strategic focus, for instance, we need to reinforce the importance of a ‘demand driven’ process. This means society would need to set these priorities. As an example, the (very) low prices for current antibiotics (among other things) has effectively sent signals to the private sector that these are not a priority. On the positive, additional incentives are currently being discussed and implemented, with a consequent increase in activity. Neglected tropical and rare diseases are two areas which reinforce that the private sector reacts to incentives. With the more novel Advanced Therapeutic Medicinal Products (ATMPs) and cell and gene therapies, there might be a risk of creating a two-tier system in Europe with only a selected group of countries having access to them. We will need to be creative and innovative to fund them and ensure wider access, including using modified or alternative evaluation models to those used for more ‘traditional’ medicines.

Additional policy options and incentives for rewarding medical innovation should go beyond prices – for a comprehensive list of ‘families’, see Mestre-Ferrandiz et al. (39) – as these are also important to direct private R&D investments. In particular, procurement policies and mechanisms from the demand side (i.e., payers) can be used smartly to drive the types of innovation needed and ensure a regular and competitive supply.

### 4.7 Need to create the environment in Europe for innovation (product, process and system) in the future; otherwise we risk losing competitiveness, if we are not losing it already

How to measure ‘competitiveness’ of a region/country is always complex, as several dimensions should be reflected. Focusing on R&D investments is a starting point, and as discussed in Section 2, arguably the core of R&D has moved away (or is moving away) from EU to the US and other regions in Asia. Clinical research is still happening in the EU, but less so than in the US, and China’s (and Asia Pacific more generally) importance is increasing. This evidence is already demonstrating that the European pharmaceutical sector is losing competitiveness with regards to these other regions. Moreover, within Europe, we see differences in competitiveness across member states, which will probably remain unless specific actions are taken. To overcome this, the bigger countries, with different offers for regional bioclusters (such as Spain, France or Germany), have the critical mass and financial investments, but we should not forget the skills and talent from EU13 countries, including an interesting SME population, and growing IT and data capabilities.

We argue Europe needs a stronger direction from the European institutions, in order to adequately incentivize innovation. For instance, the EU Orphan Medicinal Products Regulation, has been critical for the development of treatments for rare diseases, known as orphan medicinal products, with a regulatory framework providing specific incentives for pharmaceutical companies, including market exclusivity, reduced fees, and protocol assistance, to encourage research and development in this area where commercial rewards may be limited. Also, European Reference Networks (ERNs) are virtual networks of healthcare professionals and centers of expertise across Europe, aiming to facilitate the diagnosis and treatment of complex or rare diseases that require specialized knowledge and resources. By promoting knowledge exchange and collaboration, ERNs can enhance the development and delivery of innovative therapies and treatments, benefiting patients. We also hope that the EU Health Data Space initiative will help here, but this will take time.

Of course, another example illustrating the importance of a European strong direction can be seen with the creation of the European Medicines Agency (EMA), playing a crucial role in the evaluation and regulation of medicines in the EU. It ensures that pharmaceutical products meet high standards of efficacy, safety, and quality. By providing a centralized and harmonized regulatory framework, the EMA enables efficient access to new medicines across EU member states. Moreover, there are efforts from the EMA to support academics and developers with regulatory issues and quality of data by providing scientific advice and protocol assistance^6^, including also the PRIME scheme, which provides enhanced support for the development of priority medicines that target an unmet medical need, that may offer a major therapeutic advantage over existing treatments, or benefit patients without treatment options^7^.

More generally, connectivity among Directorate Generals of the EC needs to be dramatically increased (SANTE, GROW and CONNECT especially) if a truly harmonized approach to healthcare research and innovation is to be implemented – but also encouraging the expansion of public private partnerships as a model to follow (related to option IV above).

With the complexity and multidimensionality of the pharmaceutical innovation process, the creation of an innovation environment in Europe implies a number of qualities: (i) breaking silos and enabling people to work together; (ii) address dynamic uncertainties and improve flexibility; (iii) build trust among actors, and correctly prioritize; (iv) support smaller clusters, by connecting knowledge and different groups, and ensure smaller EU countries are connected with and have support of bigger countries.

### 4.8 Need to learn from ‘failure’

Given all the uncertainties in biomedical research, we need to learn from ‘failure’. We feel that it has been quickly forgotten that we had hundreds/thousands of projects in development to treat COVID-19 that did not yield specific therapies. However, this should not be seen as waste, given that research is valuable as it leads to greater knowledge and other conclusions – however, it takes sustained confidence to keep moving. The experience with medicines against Alzheimer’s Disease indeed highlights the importance of ‘failures’; with decades of resources invested, a static assessment of such investment would probably conclude it was not worth it. Nonetheless, we believe we need to take a dynamic perspective, that it is worth it, and we need to continue to push boundaries and keep the faith. Of course, governments face political pressure and financial difficulties, so they face a challenging situation that warrants the careful consideration of the ‘value’ of innovation longer term and the future focus priorities and perspective.

### 4.9 Need for major policy change: more connected and harmonized national health systems

Finally, we argue that national health systems should enhance their interconnectedness and harmonization to collectively leverage the advantages of innovation. We are in a contradictory situation where the European research infrastructure is on the one hand indeed collaborative (we have decades of EC framework programs creating an amazing cooperative research network). But on the other hand, (national) health systems have remained fragmented, perhaps for good reasons, due to the competencies and priorities not being the same (the national vs. European dichotomy mentioned in the Introduction). Nonetheless, we believe such collaborations across health systems will maximize the benefit from research networks in our teaching hospitals, translational research, and clinical research networks. A more systematic connection across the many different systems is needed. This requires guidance from stakeholders at all levels to achieve and especially support at a European policy level. We know this will be challenging; however it is important to achieve to meet the following two initial suggested objectives:

1. The demonstration of value, on all elements of the patient pathway needs to be defined. The value proposition needs to go to the relevant Ministry of Health, as they have power to move from bench to bedside. However, if this push is not harmonized at European level, it will not happen. The innovative industry needs to see clear signals to direct its R&D toward areas that society values and that improve the lives of citizens in all countries. One step in the right direction has been taken through the launch of the “Transforming health and care systems” project as part of the Horizon Europe program, a Partnership focusing on “its ambition to address and trigger global and long-term changes in the complex health and care research and innovation ecosystems. This will be achieved through a Partnership in which all stakeholders can work together to stimulate and nurture research and innovation activities”^8^.
2. The realization of the importance of prevention in healthcare is vital to attain the benefits to individuals, patients, society and economies of healthy populations. The importance of guidance on effective use of diagnostics to ensure patients access treatment in non-communicable diseases where there are effective options as well as the need to improve immunization to limit the effects of communicable diseases are two areas which require greater coordination across all stakeholders and all EU members states. These diseases know no boundaries and for improvement in population health a coordinated approach is needed.

## 5 Discussion

The aim of this paper has been to provide some reflections on the European life sciences innovation ecosystem, including what we see as key challenges and potential options to address these. It is important to understand the role of the different agents, and how to strengthen well established and emerging ecosystems in Europe through advancing innovation and optimized funding efficiency. It is critical to ascertain how the strengths of each actor can be leveraged to bring new medicines/treatments to market, quicker and more efficiently.

During 2023, the European Commission adopted a proposal for a new Directive and a new Regulation, which is proposed to revise and replace the existing general pharmaceutical legislation focussing on improving equitable access to patients across all EU member states. We do not intend to analyse the impact of such legislation, however a key element is the introduction of a linkage between regulatory data protection period and the launch and supply in all Member States. In October 2023, the Committee on the Environment, Public Health and Food Safety (ENVI) published their Draft Report on the Directive and among other changes, eliminated this link. ENVI advocates for a more flexible approach, whereby “the marketing authorisation holder shall, upon request by a Member State in which the marketing authorisation is valid, submit in good faith an application for pricing and reimbursement no later than 2 years from the date when the Member State made its request” [(40), page54], or within 4 years for SMEs and not for profit entities. Along similar lines, EFPIA companies have committed to file for pricing and reimbursement in every Member State within 2 years of marketing authorisations unless the Member State does not wish to have the medicine. Linking regulatory data protection with availability of treatments in all Member States needs further analysis, at a minimum in terms of the impact this might have on global launch strategies and incentives for R&D more generally. The process and barriers for access are multifactorial and cannot be solved through pressure on one actor in the system. Furthermore, there is risk linked to the appropriate funding of healthcare systems to support the commitment for wider access, not just in terms of financial resources but also in terms of infrastructure to assess the value of all new medicines, diagnose patients appropriately and deliver treatment. At the time of writing this paper, the Legislation was still under negotiation, although the European Parliament adopted its position in April 2024, and the New Parliament after the June 2024 European elections will need to follow it up^9^. It could be argued this Parliament position does not address adequately all the challenges raised here, and we are at a critical point to ensure the legislation can actually deliver what is intended to achieve, better access.

Moving forward, we need a trusted environment, with strategic collaborations between the public and private sectors, and policy initiatives and incentives should be focussed to strengthen the infrastructure with the aim of fostering such optimal alliances. The pros of increased collaboration include improved speed of access to novel assets, however as the system and processes become more complex, often involving multiple steps, greater clarity of pathways for all stakeholders, especially patients, is needed.

The global collaboration landscape is evolving, and the APAC region is investing heavily in building competitive pharmaceutical R&D sectors and attracting investors. This growth can influence global collaboration opportunities and competitive dynamics, with the risk that Europe’s share keeps decreasing. But we feel that our options discussed in this paper can help reverse the trend.

## Data Availability

The original contributions presented in the study are included in the article, further inquiries can be directed to the corresponding author.

## Footnotes

^1^More information can be found here: https://eic.ec.europa.eu/about-european-innovation-council_en

^2^EIT Health was established in 2015, as a ‘knowledge and innovation community’ (KIC) of the European Institute of Innovation and Technology (EIT). More information can be found here: https://eithealth.eu/who-we-are/

^3^More information can be found here: https://eithealth.eu/programmes/venture-centre-of-excellence/

^4^More information on IMI can be found in Laverty and Meulien (40).

^5^More information can be found here: https://www.who.int/europe/initiatives/who-europe-access-to-novel-medicines-platform

^6^See for instance: https://www.ema.europa.eu/en/human-regulatory-overview/research-development/scientific-advice-protocol-assistance

^7^More information on PRIME can be found here: https://www.ema.europa.eu/en/human-regulatory-overview/research-development/prime-priority-medicines

^8^More information here: https://www.thcspartnership.eu

^9^Further information on this position can be found here: https://www.europarl.europa.eu/news/en/press-room/20240408IPR20308/parliament-adopts-its-position-on-eu-pharmaceutical-reform
